# Real-world anatomical and functional outcomes following Faricimab therapy in neovascular age-related macular degeneration: a one-year observational study

**DOI:** 10.3389/fmed.2026.1901385

**Published:** 2026-09-28

**Authors:** Settimio Rossi, Carlo Gesualdo, Maria Consiglia Trotta, Raffaele Perrotta, Fabiana Anna D'Agostino, Davide Sabbatino, Claudia Gerosolima, Rosalba Casaburi, Francesca Simonelli

**Affiliations:** 1 Eye Clinic, Multidisciplinary Department of Medical, Surgical and Dental Sciences, University of Campania “Luigi Vanvitelli”, Naples, Italy; 2 Department of Life Sciences, Health and Health Professions, Link Campus University, Rome, Italy; 3 Department of Experimental Medicine, University of Campania “Luigi Vanvitelli”, Naples, Italy; 4 Eye Unit, G. Rummo Hospital, Benevento, Italy

**Keywords:** Faricimab, long-acting drugs, neovascular age-related macular degeneration, real-life outcomes, treat-and-extend

## Abstract

**Introduction:**

This study evaluated 12-month anatomical and functional outcomes of Faricimab in Italian patients with neovascular age-related macular degeneration (nAMD).

**Methods:**

30 eyes from 30 participants who completed the 12-month follow-up were retrospectively analyzed at “Luigi Vanvitelli” University Eye Clinic (Naples) and “Rummo” Hospital Ophthalmology Unit (Benevento). All underwent best-corrected visual acuity (BCVA), spectral-domain optical coherence tomography (SD-OCT), and optical coherence tomography angiography (OCTA). Patients were stratified by anti-vascular endothelial growth factor (VEGF) treatment [naïve (*N* = 22); non-naïve (*N* = 8)], baseline macular neovascularization (MNV) type and fluid distribution. BCVA and central retinal thickness (CRT) were assessed at 6 and 12 months. The association between baseline subretinal hyperreflective material (SHRM) status and longitudinal BCVA and CRT was also investigated.

**Results:**

The intravitreal injection number was similar between naïve and non-naïve eyes (median, 6 vs. 7; *p* = 0.093). One adverse event (vitreitis with ocular hypertension) occurred. Mixed-effects modelling showed significant longitudinal improvements in BCVA from baseline to months 6 and 12, with no significant time-by-treatment-history interaction. Baseline retinal fluid distribution was not significantly associated with differences in BCVA changes, while baseline SHRM status showed a significant time-by-SHRM interaction for BCVA, although multiplicity-adjusted between-group comparisons were not significant at any time point. CRT decreased significantly from baseline to months 6 and 12, whereas the change between months 6 and 12 was not statistically significant. SHRM thickness also decreased significantly from baseline to months 6 and 12. Exploratory analyses by MNV subtype did not identify significant differences in 12-month BCVA or CRT. In exploratory multivariable analysis, baseline BCVA was the only factor significantly associated with 12-month BCVA, whereas age, previous anti-VEGF treatment, and baseline SHRM were not.

**Discussion:**

This retrospective two-center study showed significant improvements in BCVA and CRT after 12 months of Faricimab, with no statistically detectable difference in longitudinal BCVA according to previous anti-VEGF exposure. Although baseline SHRM was associated with a significant time-by-SHRM interaction for BCVA, the absence of significant multiplicity-adjusted between-group differences limits interpretation of a differential response according to SHRM status. Given the small sample size and retrospective design, these findings should be interpreted cautiously.

## 1 Introduction

Anti-vascular endothelial growth factor (VEGF) drugs have been the gold standard for the treatment of neovascular age-related macular degeneration (nAMD) for more than a decade (1–3). However, despite significant improvements in the management and prognosis of nAMD, due to frequent disease reactivation with intraretinal exudation, patients require repeated intravitreal anti-VEGF injections, resulting in a significant burden for both patients and physicians and a proportionally increased risk of complications (4–7).

Furthermore, the response to anti-VEGF therapy is quite variable; in fact, many patients show an incomplete and/or failed response. In general, an analysis of the main randomized clinical trials (RCTs) shows a suboptimal response rate between 30% and 50%, and up to approximately 30% of patients are considered non-responders (8–15). Although the mechanisms underlying treatment failure have not been fully elucidated, it is reasonable to speculate that, in addition to undertreatment, genetic polymorphisms may contribute to the reduced efficacy of anti-VEGF therapy observed in some patients (16–18).

In this context, recent years have seen the development of several strategies to optimize long-term therapy with anti-VEGF, including new treatment regimens such as treat-and-extend (TAE), the use of innovative biomarkers to correctly target therapy, and finally the introduction of new long-acting drugs with innovative molecular targets (19–23).

In this regard, in January 2022, the FDA approved Faricimab (Vabysmo; Roche/Genentech, Basel, Switzerland), a novel bispecific antibody that simultaneously binds and neutralizes both angiopoietin 2 (Ang-2) and VEGF-A, making it a unique therapeutic option for the treatment of nAMD, and macular edema associated with diabetes (DME), or retinal vein occlusion (RVO). The dual inhibition of Ang-2 and VEGF-A may promote vascular stability and reduce vascular hyperpermeability, providing a potential mechanistic rationale for improved disease control and treatment durability (24, 25). This mechanism of action may translate into greater treatment durability compared to therapies that target the VEGF pathway alone. Indeed, the pivotal TENAYA and LUCERNE studies demonstrated that in treatment-naive eyes with nAMD, Faricimab administered every 8–16 weeks achieved anatomical and functional outcomes comparable to those obtained with Aflibercept administered every 8 weeks. Furthermore, Faricimab showed the potential to extend the interval between two consecutive injections up to 16 weeks in almost 50% of eyes (26). Preliminary data from several real-world studies also support the efficacy of Faricimab, showing stabilization of best-corrected visual acuity (BCVA) and effective control of retinal fluid (27–29). However, to date, very few Italian real-world studies have examined the anatomical and functional outcomes of Faricimab in the medium to long term. Therefore, the aim of the present study was to analyze the 1-year real-world outcomes observed after initiation of intravitreal Faricimab therapy in patients with active nAMD, both naïve and non-naïve, and to correlate the obtained anatomical-functional outcomes with structural biomarkers (30–32).

## 2 Materials and methods

This is a retrospective observational study in which 30 patients (30 eyes) diagnosed with active nAMD were enrolled and followed both at the Eye Clinic of the University of Campania “Luigi Vanvitelli” in Naples (Italy), and at the Ophthalmology Unit of the “Rummo” Hospital in Benevento, Italy, for a period of one year (from June 2024 to July 2025). Table 1 summarizes the demographic and clinical data of the included subjects. The study adhered to the principles outlined in the Declaration of Helsinki and was approved by the Ethics Committee “Luigi Vanvitelli” University (Protocol number 0013692/i, 09-05-2024). Informed consent to participate in the study was obtained from all participants. Inclusion criteria were: age 50 years or older; patients with at least 12 months of follow-up; subfoveal, juxtafoveal, or extrafoveal macular neovascularization (MNV), with signs of macular exudation confirmed by spectral-domain optical coherence tomography (SD-OCT) and OCT angiography (OCTA); any type of AMD-related MNV as already widely described in the literature (33):

- MNV type 1 (occult): characterized by a sub- retinal pigment epithelium (RPE) fibrovascular complex arising from the choriocapillaris into the sub-RPE space.
- MNV type 2 (classic): characterized by the presence of hyperreflective material originating from the choriocapillaris, penetrating through the RPE and spreading subretinally.
- MNV type 3 (RAP): characterized by proliferating vessels that extend from the deep retinal capillary plexus towards the outer retina.

**Table 1:** Demographic and clinical ophthalmological characteristics of the enrolled patients at baseline.

Characteristics	Total cohort	Naïve	Non-naïve
Age; years (mean ± SD)	75.1 ± 7.8	75 ± 8.7	76 ± 4.7
Gender; number (%)	Male: 10 (33.3%)	7 (31.8%)	3 (37.5%)
	Female: 20 (66.7%)	15 (68.2%)	5 (62.5%)
Systemic risk factors			
Hypertension; number (%)	24 (80%)	18 (82%)	6 (75%)
Smoking; number (%)	18 (60%)	13 (59%)	5 (62%)
Alcohol; number (%)	5 (17%)	4 (18%)	1 (12.5%)
Eyes; number (%)	30 (100%)	22 (73.3%)	8 (26.7%)
Lens status; number (%)	Phakic: 12 (40%)	9 (41%)	3 (37.5%)
	Pseudophakic: 18 (60%)	13 (59%)	5 (62.5%)
MNV type; number (%)	Type 1 (*n*. 13) (43.3%)	11 (50%)	2 (25%)
	Type 2 (*n*. 15) (50.0%)	9 (41%)	6 (75%)
	Type 3 (*n*. 2) (6.7%)	2 (9%)	0
SHRM; number (%)	SHRM+ eyes: 14 (46.7%)	12 (54.6%)	2 (25%)
	SHRM− eyes: 16 (53.3%)	10 (45.4%)	6 (75%)
Baseline BCVA; L	40.5 ± 19.1	50.5 (28.5–55)	30.5 (20–51.2)
Baseline CRT; µm	372 ± 130	374 (278–455)	300 (288–354)

Continuous variables are reported as mean ± SD or median (IQR), according to distribution. BCVA, best-corrected visual acuity; CRT, central retinal thickness; L, letters; MNV, macular neovascularization; SHRM, subretinal hyperreflective material; SHRM+/−, eyes with SHRM presence/absence.

We adopted the following exclusion criteria: previous treatments with Faricimab; concomitant ocular diseases such as diabetic retinopathy, hereditary retinal dystrophies, retinal vascular occlusions, uveitis; laser photocoagulation, intravitreal steroid injections and/or vitrectomy performed in the study eye within the last 6 months. Patients with media opacity (e.g., clinically significant lens opacity) that prevented proper fundus visualization were also excluded.

Patients were retrospectively identified from the institutional databases of the two participating centers. All consecutive eligible patients meeting the inclusion criteria were included, while patients who had not completed 12 months of follow-up were excluded.

### 2.1 Study design

We divided the entire cohort into two groups: Group 1 (treatment-naïve eyes) and Group 2 (non-naïve eyes), both of which underwent a loading dose of 4 monthly intravitreal Faricimab injections (loading dose) plus a “Treat and extend” (TAE) regimen with treatment intervals extended to Q8w, Q12w, or Q16w according to disease activity. All participants underwent comprehensive ophthalmological evaluation, including: BCVA assessment using Early Treatment Diabetic Retinopathy Study (ETDRS) chart at 4m; anterior segment biomicroscopy and binocular indirect ophthalmoscopy performed using a Haag-Streit slit lamp; Spectral-Domain Optical Coherence Tomography (SD-OCT) and Angiography-OCT (OCTA) performed at baseline, 6 and 12 months. SD-OCT imaging was conducted using Cirrus 6000 (Carl Zeiss Meditec, Dublin, CA, USA), employing the protocol Macular Cube 512 × 128. Central retinal thickness (CRT) was measured as the average retinal thickness within a 1 mm-diameter circle centered on the fovea.

### 2.2 Study outcomes

The primary outcome of the study was the change in BCVA from baseline to the 12-month follow-up visit. The change in CRT over the same period was defined as a key secondary anatomical outcome, complementary to the primary functional endpoint. Secondary outcomes included changes in BCVA and CRT at 6 months, the achievement of extended treatment intervals, and the occurrence of ocular and systemic adverse events during the 12-month follow-up period. Secondly, patients were stratified according to anti-VEGF treatment status (naïve vs. non-naïve), baseline MNV subtype and baseline fluid distribution (intraretinal [IR], subretinal [SR], and sub-RPE [SRPE]), to evaluate the changes in BCVA during follow-up in each analyzed subgroup. Baseline retinal fluid distribution was recorded according to the presence of IRF, SRF, and SRPE. Because different fluid compartments could coexist within the same eye, these categories were not mutually exclusive. Finally, we evaluated the influence of a new OCT biomarker such as subretinal hyperreflective material (SHRM) on BCVA and its anatomical evolution during the 12- month follow up, as already described (34, 35). Briefly, SHRM thickness was measured from the inner border of the hyper-reflective lesion to the inner surface of the RPE, when identifiable, or to Bruch's membrane in cases of RPE disruption or absence. All B-scans included in the macular cube acquisition (512 × 128 protocol, 6 × 6 mm scan area) were independently reviewed by graders (R.C. and C.G.) to identify and quantify the maximum SHRM thickness within the entire macular volume. Intergrader reliability of SHRM thickness measurements was assessed using the intraclass correlation coefficient (ICC). Measurements obtained independently by the two graders (R.C. and C.G.) before any adjudication were used for the reliability analysis. A total of 42 paired measurements were available, corresponding to 14 eyes evaluated at baseline, month 6, and month 12. A two-way random-effects model assessing absolute agreement for single measurements (ICC[2,1]) was applied, and the ICC estimate with its 95% confidence interval (CI) was reported. Given the exploratory nature of this analysis and the limited number of eyes, the 42 paired eye-visit measurements were pooled for the ICC analysis. Because multiple measurements were obtained from the same eye, these observations were not statistically independent; therefore, this approach was interpreted as an assessment of intergrader agreement across paired eye-visit measurements rather than as a longitudinal reliability model accounting for within-eye clustering. For subsequent analyses, the mean value of the two independent grader measurements was used as the representative SHRM thickness value. In cases of marked disagreement between graders, the corresponding B-scans were subsequently reviewed jointly with a senior retinal specialist (S.R.) for confirmation of the measurement; these adjudicated values were not used for the intergrader reliability analysis.

### 2.3 Statistical analysis

Data distribution was assessed using the Shapiro–Wilk test, and homogeneity of variances was assessed using Levene's test. These tests were used as descriptive diagnostics and were not considered sole criteria for selecting longitudinal statistical models, given the repeated-measures structure of the ophthalmological data.

All statistical analyses were performed using R (version 4.6.1; R Foundation for Statistical Computing, Vienna, Austria) and IBM SPSS Statistics for Windows, Version 30.0 (IBM Corp., Armonk, NY, USA). Linear mixed-effects models (LMMs) were fitted in R using the lme4 and lmerTest packages with restricted maximum likelihood (REML) estimation. Kenward–Roger degrees-of-freedom adjustment was used for inference on fixed effects and Type III omnibus tests. Estimated marginal means (EMMs) and pairwise contrasts were obtained using the emmeans package, with degrees of freedom estimated using the Kenward–Roger method. Model outputs were extracted and formatted using broom.mixed. IBM SPSS Statistics was used for descriptive analyses, non-parametric tests, multivariable linear regression, regression diagnostics, and intraclass correlation coefficient (ICC) assessment. Intergrader reliability was assessed using the ICC based on a two-way random-effects model for absolute agreement and single measurements [ICC(2,1)].

Continuous variables were reported as mean ± standard deviation (SD) or median (interquartile range, IQR), as appropriate, whereas categorical variables were presented as absolute numbers and percentages. Baseline characteristics were summarized descriptively. No formal hypothesis testing was performed to compare baseline characteristics or subgroup sizes between treatment groups, given the retrospective and non-randomized study design.

The primary longitudinal endpoint was BCVA change from baseline to month 12, while the longitudinal analysis included BCVA measurements at baseline, month 6, and month 12. Change in CRT from baseline to month 12 was prespecified as the key secondary anatomical outcome. Additional secondary outcomes included longitudinal changes in BCVA and CRT at month 6 and the occurrence of ocular and systemic adverse events during the 12-month follow-up period. Given the retrospective, real-world, and exploratory nature of the study, secondary and exploratory analyses were interpreted as hypothesis-generating rather than confirmatory.

#### 2.3.1 Primary longitudinal analyses

Longitudinal changes in BCVA and CRT were assessed using LMMs with REML estimation to account for the correlation between repeated measurements obtained from the same eye and to model within-eye variability. Time was included as a categorical fixed effect (baseline, month 6, and month 12), and eye identification was included as a random intercept. Baseline was specified as the reference category for time.

To investigate whether baseline SHRM status was associated with longitudinal BCVA or CRT trajectories, baseline SHRM status and the time-by-SHRM interaction were additionally included as fixed effects, with SHRM-negative status specified as the reference category. The overall effects of time, SHRM status, and the time-by-SHRM interaction were assessed using Type III omnibus tests with Kenward–Roger degrees-of-freedom adjustment. The time-by-SHRM interaction was used to assess whether longitudinal trajectories differed according to baseline SHRM status. Fixed-effect coefficients, standard errors, degrees of freedom, and corresponding *p*-values were obtained from the fitted LMMs. Coefficient-level 95% confidence intervals for fixed effects were calculated using the same Kenward–Roger degrees-of-freedom framework as that used for the corresponding two-sided coefficient-level *p*-values, to ensure consistency between confidence intervals and hypothesis tests. EMMs and pairwise contrasts were used to estimate changes between time points and differences between SHRM groups, with 95% confidence intervals and multiplicity-adjusted *p*-values reported for the corresponding sets of pairwise comparisons. Tukey adjustment was used for pairwise comparisons among time-point EMMs in the overall BCVA analysis, Sidak adjustment was used for the corresponding overall CRT comparisons, and Holm adjustment was used for within-group longitudinal comparisons and for between-group comparisons performed separately at each time point.

#### 2.3.2 Treatment-history analysis

To investigate whether previous anti-VEGF treatment was associated with differences in longitudinal BCVA and CRT trajectories, an additional LMM was fitted including time, treatment history (treatment-naïve vs. non-naïve), and the time-by-treatment-history interaction as fixed effects, with eye identification included as a random intercept. This analysis was considered exploratory and hypothesis-generating. Exploratory within-group EMM comparisons were performed where relevant and adjusted for multiple comparisons using Holm's method within the corresponding comparison family. Between-group EMM contrasts comparing treatment-naïve and non-naïve eyes at baseline, month 6, and month 12 were treated as a single family of three comparisons and adjusted jointly using Holm's method.

#### 2.3.3 Model diagnostics

LMM assumptions were evaluated using residual diagnostics, including assessment of residual distribution, linearity, homoscedasticity, and potentially influential observations. For the LMMs, Cook's distance was inspected descriptively as a screening measure of potentially influential observations, without using a predefined exclusion threshold.

#### 2.3.4 Exploratory SHRM analysis

SHRM thickness, available in 14 eyes, was assessed longitudinally across baseline, month 6, and month 12 using the Friedman rank-sum test. When the omnibus test was significant, pairwise comparisons were performed using Wilcoxon signed-rank tests with Holm adjustment for multiple comparisons. These analyses were considered exploratory because of the limited sample size and the non-normal distribution of SHRM thickness.

#### 2.3.5 Additional exploratory analyses

Baseline anatomical characteristics, including MNV subtype, retinal fluid compartments, and CRT, were evaluated in relation to functional and anatomical outcomes. Differences in 12-month BCVA and CRT among MNV subtypes were assessed using the Kruskal–Wallis test, followed by Dunn's *post hoc* test when appropriate. Intraretinal (IR), subretinal (SR), and sub-retinal pigment epithelium (SRPE) fluid compartments were evaluated independently according to their presence or absence at baseline, as these compartments were not mutually exclusive. The change in BCVA from baseline to month 12 was compared between eyes with and without each fluid compartment using the Mann–Whitney *U*-test. The association between baseline CRT and BCVA at month 12 was assessed using Spearman's correlation analysis. The number of intravitreal injections administered during follow-up was compared between treatment-naïve and non-naïve eyes using the Mann–Whitney *U*-test as an exploratory measure of treatment exposure. Confidence intervals for the Q16w proportion were calculated using the Wilson score method.

#### 2.3.6 Exploratory regression analysis

A multivariable linear regression model was constructed to investigate baseline factors potentially associated with functional outcome, with BCVA at month 12 as the dependent variable. Independent variables were selected *a priori* based on clinical relevance and included baseline BCVA, age, anti-VEGF treatment status (0 = treatment-naïve; 1 = non-naïve), and baseline SHRM presence (0 = absent; 1 = present). Model coefficients (*β*), 95% confidence intervals (CIs), *p*-values, coefficient of determination (*R*^2^), and adjusted *R*^2^ were reported.

Regression assumptions were evaluated by inspection of residual plots and assessment of linearity, residual normality, and homoscedasticity. Influential observations were assessed using Cook's distance, and multicollinearity was evaluated using the variance inflation factor (VIF). The conventional screening threshold of 4/n was used to identify potentially influential observations. Given the limited sample size and the identification of a potentially influential observation exceeding this threshold, a sensitivity analysis was performed by excluding the observation with the highest Cook's distance and repeating the multivariable linear regression model. The consistency of regression coefficients, 95% CIs, and statistical significance of predictors was assessed between the original and sensitivity models.

#### 2.3.7 Multiplicity and statistical significance

For longitudinal LMM analyses, multiplicity adjustment was applied to EMM pairwise comparisons within the corresponding sets of comparisons. Coefficient-level *p*-values reported in the Supplementary Tables represent unadjusted model-based tests of individual reference-category contrasts and were not interpreted as omnibus tests of the corresponding factors. For factors represented by multiple model coefficients, overall statistical significance was assessed using the corresponding Type III omnibus test. For the overall longitudinal analyses, Tukey adjustment was used for pairwise comparisons among time-point EMMs for BCVA, whereas Sidak adjustment was used for the corresponding CRT comparisons. For the treatment-history analyses, exploratory within-group EMM comparisons were adjusted using Holm's method within the corresponding comparison family, whereas the three between-group comparisons between treatment-naïve and non-naïve eyes at baseline, month 6, and month 12 were treated as a single comparison family and adjusted jointly using Holm's method. For SHRM-related LMM analyses, Holm adjustment was used for within-group longitudinal comparisons, whereas the three between-group comparisons between SHRM+ and SHRM− eyes at baseline, month 6, and month 12 were treated as a single comparison family and adjusted jointly using Holm's method. All subgroup and treatment-history analyses were considered exploratory and hypothesis-generating rather than confirmatory.

A two-sided *p*-value <0.05 was considered statistically significant.

## 3 Results

30 eyes from 30 consecutive patients with nAMD were retrospectively analyzed; all included eyes had complete data at baseline, month 6, and month 12, according to the study eligibility criteria. Among patients initially identified from institutional databases, 5 were excluded because 12-month follow-up data were unavailable and 8 because of significant media opacity. The final study population included 10 males (33.3%) and 20 females (66.7%). No included patients had missing visits during the 12-month observation period. There were 22 treatment-naïve eyes and 8 non-naïve eyes. All non-naïve patients had previously been treated with intravitreal Aflibercept (2 mg), for a mean treatment duration of 6.5 ± 0.5 years. The switch to Faricimab was prompted by decreased treatment response and/or the progressive shortening of treatment intervals required to maintain disease control. The mean age of the study population was 75.1 ± 7.8 years. Notably, no substantial age distinctions were highlighted between the two subgroups (naïve vs. non-naïve). The number of intravitreal injections administered during follow-up was 6 (IQR, 5–7) in naïve eyes and 7 (IQR, 6.75–7) in non-naïve eyes. The number of injections did not differ significantly between groups (Mann–Whitney *U* = 54.0, *p* = 0.093). In the entire patient cohort, 19 out of 30 eyes (63.3%, 95% CI: 45.51–78.13%) reached and maintained Q16w, defined as extension to a 16-week treatment interval after loading phase with maintenance until month 12. Among naïve eyes, 14/22 (63.6%) maintained a Q16w regimen, receiving up to two IVT injections after the initial loading dose until the end of follow-up. We observed only one ocular adverse reaction, characterized by vitreitis with ocular hypertension, subsequently managed with pars plana vitrectomy. No systemic adverse events were reported during the study.

Regarding the baseline MNV type, eyes were classified as follows: 43% MNV type 1 (*n* = 13); 50% MNV type 2 (*n* = 15); 7% MNV type 3 (*n* = 2). A comprehensive overview of the baseline functional and morphological characteristics of the entire cohort is summarized in Table 1.

### 3.1 BCVA analysis

The analysis of visual outcomes in the entire cohort using a LMM showed significant longitudinal changes in BCVA during follow-up. EMM BCVA values were 40.5 ETDRS letters (L) (95% CI: 33.8–47.2) at baseline, 47.6 L (95% CI: 40.9–54.3) at month 6, and 47.2 L (95% CI: 40.5–53.9) at month 12. Multiplicity-adjusted pairwise comparisons of EMMs using the Tukey method showed a significant improvement in BCVA from baseline to month 6 (+7.10 letters; 95% CI, 3.68–10.52; *p* < 0.0001) and from baseline to month 12 (+6.67 letters; 95% CI, 3.25–10.08; *p* < 0.0001) (Figure 1A). These estimates and confidence intervals are based on multiple-adjusted pairwise EMM contrasts and therefore differ from the unadjusted fixed-effect coefficients reported in Supplementary Table 1. Thus, BCVA was significantly higher at both month 6 and month 12 than at baseline.

**Figure 1:**
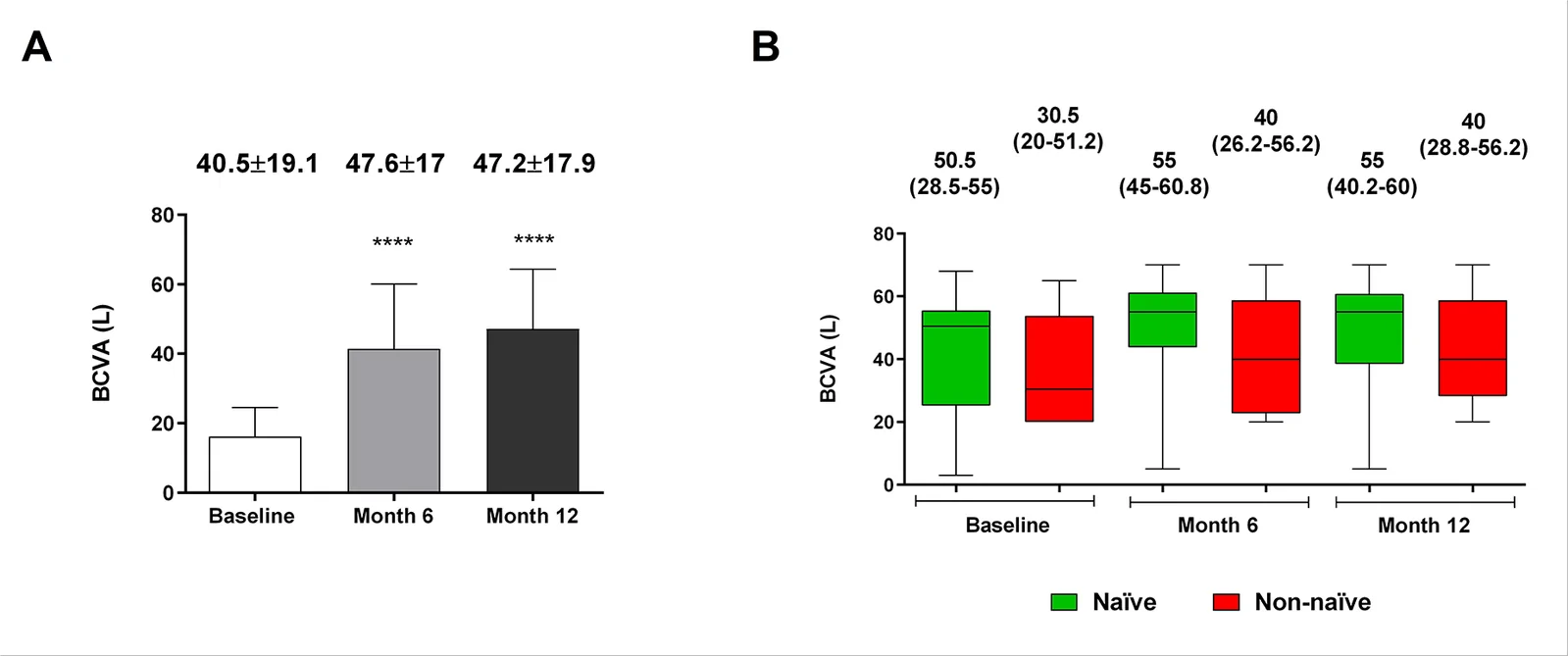
**(A)** Bar chart showing observed BCVA values (L) in the entire nAMD cohort (*N* = 30) at baseline, month 6, and month 12; **** *p* < 0.0001 for month 6 and month 12 vs. baseline, based on Tukey-adjusted pairwise contrasts of EMMs derived from the LMM. Bars represent observed mean ± standard deviation (SD). **(B)** Box-and-whisker plot illustrating observed BCVA values (L) in naïve vs. non-naïve groups (*N* = 22 vs. 8, respectively) at Baseline, Month 6, and Month 12. No statistically significant difference in longitudinal BCVA trajectories was observed between groups according to the LMM including time, treatment history, and the time-by-treatment-history interaction. Descriptive group data are presented without multiplicity-adjusted between-group comparisons. Data are presented as median and interquartile range (IQR). BCVA, best-corrected visual acuity; EMMs, estimated marginal means; L, letters; LMM, linear mixed-effects model.

An additional LMM including time, anti-VEGF treatment status (naïve vs. non-naïve), and the time-by-treatment-status interaction was performed to assess whether previous anti-VEGF exposure influenced BCVA. The model showed no statistically detectable difference in longitudinal BCVA trajectories according to previous anti-VEGF treatment status. The overall time-by-treatment-history interaction was not statistically significant (F(2, 56) = 0.300, *p* = 0.742). The corresponding fixed-effect estimates, including the treatment-history effect and time-by-treatment-history interaction terms, are reported in Supplementary Table 2A, while model-estimated BCVA values according to anti-VEGF treatment status are reported as EMMs in Supplementary Table 2B. Holm-adjusted pairwise comparisons of EMMs between treatment-naïve and non-naïve eyes, with the three time-point contrasts treated as a single comparison family, showed no statistically significant differences at baseline (estimated difference, +5.62 L; 95% CI, −9.58 to 20.80; Holm-adjusted *p* = 0.880), month 6 (+7.98 L; 95% CI, −7.23 to 23.20; Holm-adjusted *p* = 0.880), or month 12 (+6.02 L; 95% CI, −9.19 to 21.20; Holm-adjusted *p* = 0.880). For descriptive purposes, observed median BCVA values (IQR) at month 6 [55 (45–60.8) L in naïve eyes vs. 40 (26.2–56.2) L in non-naïve eyes] and month 12 [55 (40.2–60) L vs. 40 (28.8–56.2) L, respectively] are reported (Figure 1B).

Exploratory analyses according to baseline MNV subtype did not identify statistically significant differences in 12-month BCVA among MNV type 1, type 2, and type 3 (Kruskal–Wallis test, *p* > 0.05). However, these analyses were limited by the small number of eyes within each subgroup, particularly for type 3 MNV (*n* = 2), and should therefore be interpreted as hypothesis-generating rather than as evidence of comparable 12-month functional outcomes among MNV subtypes. Similarly, exploratory analyses according to baseline retinal fluid distribution did not reveal statistically significant differences in BCVA change from baseline to 12 months between eyes with and without IR, SR, or SRPE (all *p* > 0.05). IR, SR, and SRPE were evaluated independently because the fluid compartments were not mutually exclusive, and individual eyes could present with more than one fluid compartment at baseline. Given the limited subgroup sizes, these findings should be interpreted cautiously and considered hypothesis-generating.

### 3.2 CRT evaluation

The analysis of anatomical outcomes in the entire cohort using a LMM showed significant longitudinal changes in CRT during follow-up. All 30 eyes contributed data at baseline, month 6, and month 12. Multiplicity-adjusted pairwise comparisons of EMMs using the Sidak method showed a significant reduction in CRT from baseline to month 6 (−130.0 µm; 95% CI: −173 to −86.3; *p* < 0.0001) and from baseline to month 12 (−112.0 µm; 95% CI: −156 to −68.6; *p* < 0.0001), whereas the change from month 6 to month 12 was not significant (−17.7 µm; 95% CI: −63.4 to 27.9; *p* = 0.621) (Figure 2A). The reported confidence intervals for the baseline-to-month 6 and baseline-to-month 12 comparisons are based on multiplicity-adjusted pairwise EMM contrasts and therefore differ from the unadjusted fixed-effect coefficient confidence intervals reported in Supplementary Table 3.

**Figure 2:**
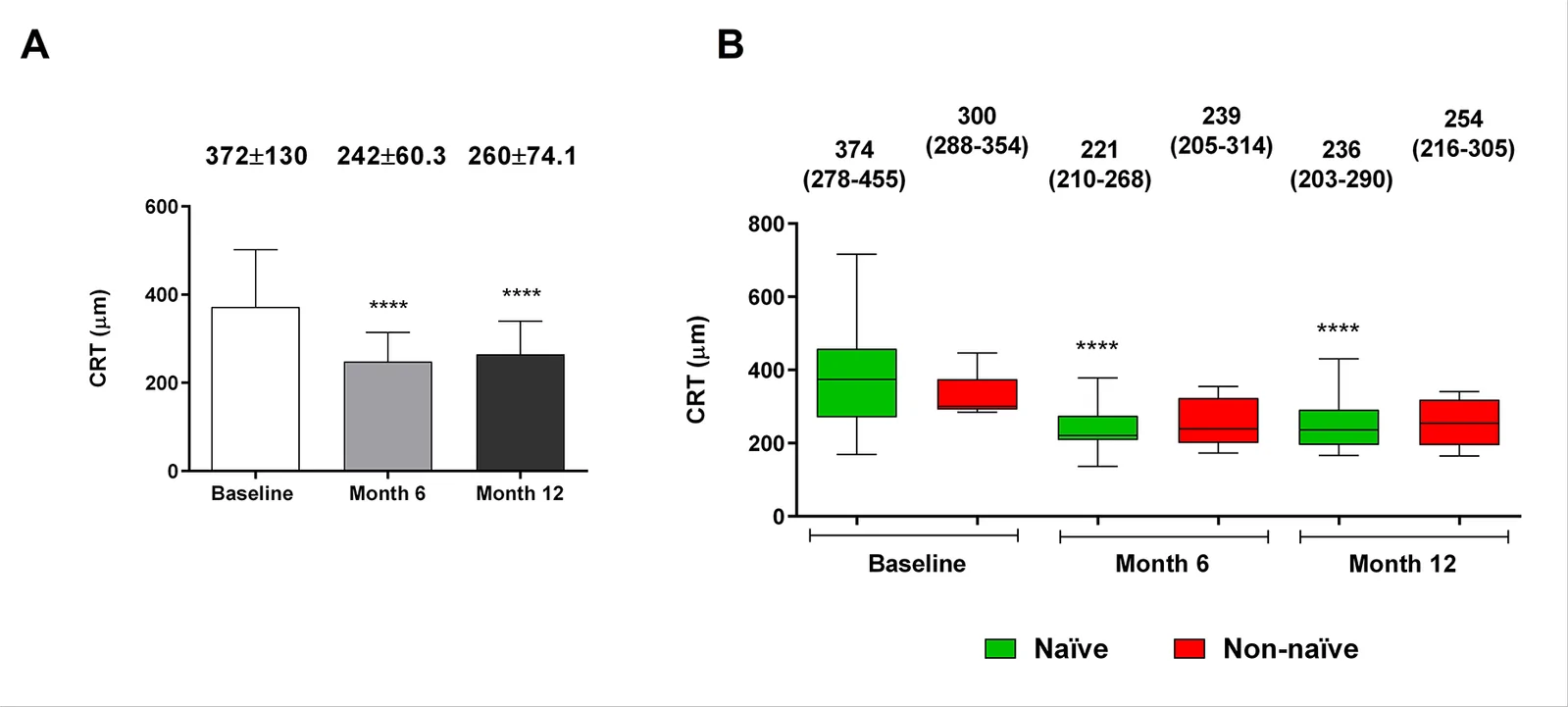
**(A)** Bar chart showing observed CRT values (µm) in the entire nAMD cohort (*N* = 30) at baseline, month 6, and month 12; **** *p* < 0.0001 for month 6 and month 12 versus baseline, based on Sidak-adjusted pairwise contrasts of EMMs derived from the LMM. Bars represent observed mean ± standard deviation (SD). **(B)** Box-and-whisker plot illustrating CRT values in naïve vs. non-naïve groups (*N* = 22 vs. 8, respectively) at Baseline, Month 6, and Month 12. No statistically significant difference in longitudinal CRT trajectories was observed between groups according to the LMM including time, treatment history, and the time-by-treatment-history interaction. Exploratory within-group comparisons in naïve eyes were performed using pairwise contrasts of estimated marginal means from the LMM, with Tukey adjustment for multiple comparisons (**** *p* < 0.0001 vs. Baseline for both Month 6 and Month 12). These comparisons were exploratory and should not be interpreted as evidence of a differential treatment response between groups. Box plots display observed data as median and interquartile range (IQR). CRT: central retinal thickness; EMMs: estimated marginal means; LMM: linear mixed-effects model.

To determine whether anti-VEGF treatment status influenced the anatomical response, an LMM including time, treatment history, and the time-by-treatment-history interaction was performed. The overall time-by-treatment-history interaction was not statistically significant (F(2, 56) = 1.887, *p* = 0.161), indicating no statistically detectable difference in longitudinal CRT trajectories according to previous anti-VEGF treatment status. The unadjusted coefficient-level tests for the month 6 × treatment-history and month 12 × treatment-history interactions were also not statistically significant (*p* = 0.063 and *p* = 0.193, respectively). Supplementary Table 4A presents the treatment-history model, including the treatment-history effect and time-by-treatment-history interaction terms; corresponding model-estimated CRT values according to anti-VEGF treatment status are reported as EMMs in Supplementary Table 4B. Exploratory within-group analyses showed a significant CRT reduction in naïve eyes (*n* = 22), from an EMM of 388 μm (95% CI: 349–428) at baseline to 237 μm (95% CI: 197–277) at month 6 and 261 μm (95% CI: 222–301) at month 12 (Holm-adjusted *p* < 0.0001 for both comparisons with baseline). For descriptive purposes, median (IQR) CRT values in naïve eyes were 374 (278–455) μm at baseline, 221 (210–268) μm at month 6, and 236 (203–290) μm at month 12 (Figure 2B). However, these within-group comparisons should be interpreted cautiously, as the overall time-by-treatment-history interaction was not statistically significant. Therefore, these exploratory within-group findings do not imply a differential treatment effect between naïve and non-naïve eyes.

Exploratory analyses according to baseline MNV subtype did not identify statistically significant differences in 12-month CRT among MNV type 1, type 2, and type 3 (Kruskal–Wallis test, *p* > 0.05). Although eyes with type 2 MNV showed a numerically greater reduction in CRT, this descriptive finding was not statistically tested as a change from baseline. Given the limited number of eyes in each subgroup, especially type 3 MNV (*n* = 2), these findings should be considered exploratory and interpreted as the absence of a statistically detectable association within this cohort rather than evidence of equivalent anatomical responses among MNV subtypes.

### 3.3 SHRM changes

SHRM was identifiable and measurable in 14/30 eyes which constituted the SHRM+ group; the remaining 16 eyes were classified as SHRM−. In the exploratory pooled analysis, intergrader agreement across pooled paired eye-visit measurements (baseline, month 6, and month 12) was high (ICC[2,1] = 0.984; 95% CI, 0.966–0.992; F(41,41) = 146.32; *p* < 0.001).

Exploratory within-group analysis of SHRM thickness showed a significant overall difference across the three time points (Friedman *χ*^2^(2) = 14.39, *p* < 0.001). Median SHRM thickness decreased from 160 (79–500) μm at baseline to 58.5 (0–293) μm at month 6 and 71.5 (0–273) μm at month 12 (Figure 3A). Pairwise *post-hoc* comparisons using Wilcoxon signed-rank tests with Holm adjustment showed significant reductions from baseline to month 6 (*p* = 0.009) and from baseline to month 12 (*p* = 0.009), whereas the difference between month 6 and month 12 was not significant (*p* = 0.398). These within-group comparisons were considered exploratory, as they were not intended to assess differences between SHRM subgroups.

**Figure 3:**
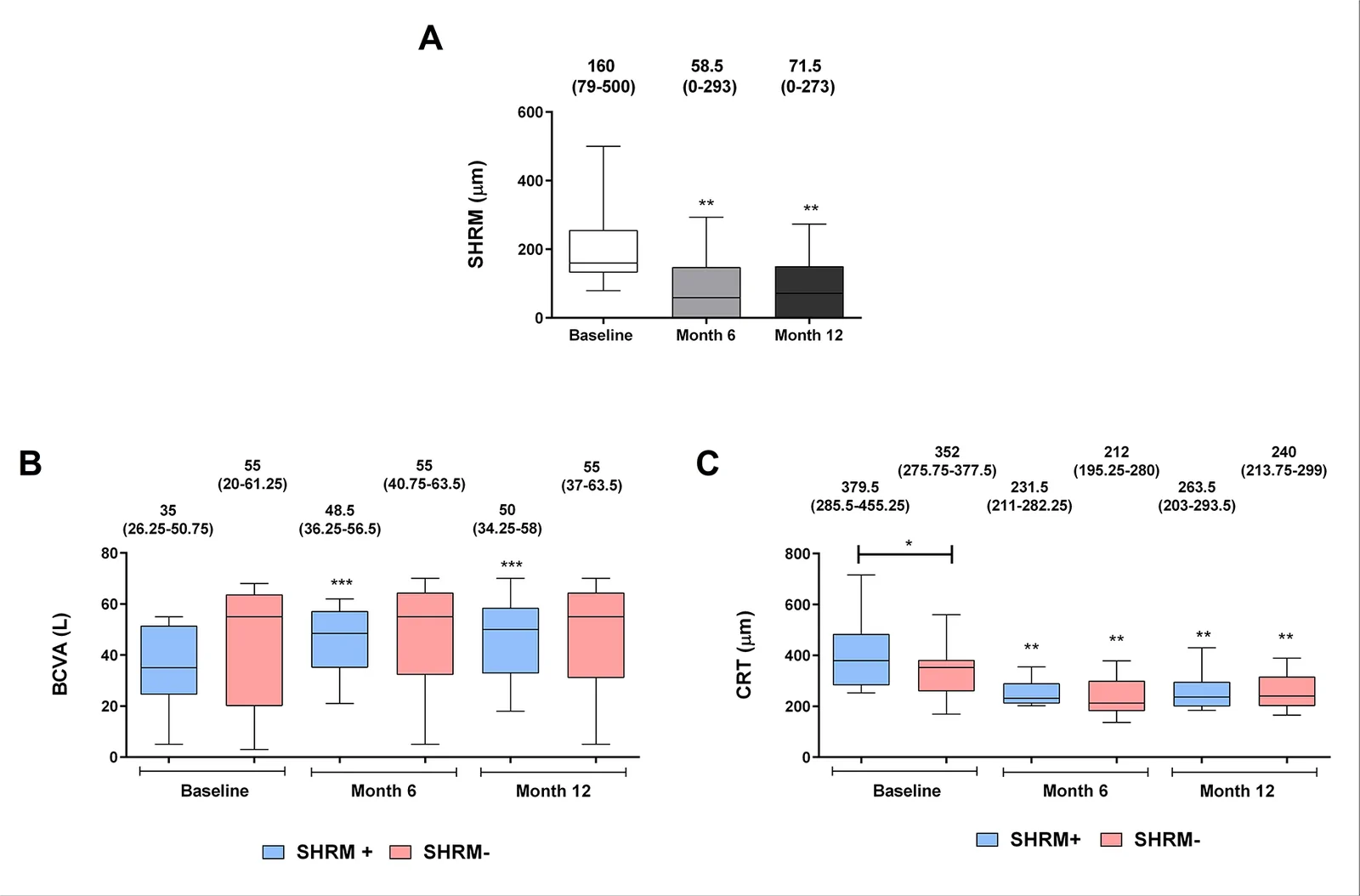
**(A)** Box-and-whisker plot showing exploratory longitudinal changes in SHRM thickness (µm) at baseline, month 6, and month 12 (*N* = 14); Friedman test: *χ*^2^(2) = 14.39, *p* < 0.001; pairwise Wilcoxon signed-rank tests with Holm adjustment: *p* < 0.01 for both baseline vs. month 6 and baseline vs. month 12. Data are presented as median and interquartile range (IQR). **(B)** Box-and-whisker plot illustrating observed BCVA values (L) in eyes with or without SHRM (SHRM+ and SHRM−, respectively) at Baseline, Month 6, and Month 12 (SHRM+: *N* = 14; SHRM−: *N* = 16). Longitudinal BCVA trajectories were evaluated using an LMM including time, baseline SHRM status, and the time-by-SHRM interaction. The overall time-by-SHRM interaction was statistically significant (Type III test, *p* = 0.040); however, Holm-adjusted between-group EMM comparisons at individual time points were not statistically significant. Holm-adjusted within-group comparisons showed significant BCVA improvement from baseline to month 6 and month 12 in SHRM+ eyes (both *p* < 0.001 vs Baseline), whereas the corresponding changes in SHRM− eyes were not statistically significant after multiplicity adjustment. Data are presented as median and IQR. **(C)** Box-and-whisker plot illustrating observed CRT values (µm) in eyes with or without SHRM (SHRM+ and SHRM−, respectively) at Baseline, Month 6, and Month 12 (SHRM+: *N* = 14; SHRM−: *N* = 16). The overall effect of time was significant (Type III test, *p* < 0.001), whereas neither the overall effect of SHRM status nor the time-by-SHRM interaction was statistically significant. Holm-adjusted within-group comparisons showed significant CRT reductions from baseline to month 6 and month 12 in both groups (all *p* < 0.01), with no significant change between Month 6 and Month 12. BCVA, best-corrected visual acuity; CRT, central retinal thickness; EMMs, estimated marginal means; L, letters; LMM, linear mixed-effects model; SHRM, subretinal hyperreflective material.

The potential association between baseline SHRM status and longitudinal visual outcomes was evaluated using a LMM including time, SHRM status, and the time-by-SHRM interaction as fixed effects, with eye identification included as a random intercept. For BCVA, the model showed a significant overall effect of time (Type III test, *p* < 0.001) and a significant time-by-SHRM interaction (Type III test, *p* = 0.040), whereas the overall effect of baseline SHRM status was not statistically significant (*p* = 0.522) (Supplementary Table 5). EMM BCVA values at baseline, month 6, and month 12 were 44.4 L (95% CI: 35.1–53.6), 48.7 L (95% CI: 39.4–57.9), and 48.1 L (95% CI: 38.8–57.3) in SHRM− eyes, and 36.1 L (95% CI: 26.2–46.0), 46.4 L (95% CI: 36.5–56.2), and 46.1 L (95% CI: 36.3–56.0) in SHRM+ eyes, respectively Holm-adjusted within-group contrasts showed no statistically significant BCVA improvement from baseline to month 6 or month 12 in SHRM− eyes (*p* = 0.0742 and *p* = 0.1068, respectively), whereas significant improvement was observed in SHRM+ eyes from baseline to month 6 (*p* < 0.0001) and month 12 (*p* < 0.0001). Holm-adjusted pairwise comparisons of EMMs between SHRM+ and SHRM− eyes, with the three time-point contrasts treated as a single comparison family, showed no statistically significant differences at baseline (estimated difference, −8.30 L; 95% CI, −21.8 to 5.23; Holm-adjusted *p* = 0.661), month 6 (−2.33 L; 95% CI, −15.9 to 11.21; Holm-adjusted *p* = 1.000), or month 12 (−1.92 L; 95% CI, −15.5 to 11.62; Holm-adjusted *p* = 1.000). These findings indicate that although the omnibus time-by-SHRM interaction was statistically significant, the multiplicity-adjusted pairwise comparisons did not demonstrate significant between-group differences at individual time points, and the significant within-group improvement observed in SHRM+ eyes should not be interpreted as evidence of a superior functional response compared with SHRM− eyes. Therefore, the interaction should be interpreted cautiously, particularly given the exploratory nature of the analysis and the limited sample size (Figure 3B; Supplementary Table 5).

The potential association between baseline SHRM status and longitudinal CRT changes was evaluated using a LMM including time, SHRM status, and the time-by-SHRM interaction as fixed effects, with eye identification included as a random intercept. For CRT, the model showed a significant overall effect of time (Type III test, *p* < 0.001), whereas neither the overall effect of baseline SHRM status (*p* = 0.294) nor the time-by-SHRM interaction (*p* = 0.128) was significant (Supplementary Table 6). EMM CRT values at baseline, month 6, and month 12 were 338 μm (95% CI: 292–384), 237 μm (95% CI: 191–283), and 259 μm (95% CI: 213–305) in SHRM− eyes, and 410 μm (95% CI: 361–460), 248 μm (95% CI: 198–297), and 260 μm (95% CI: 211–310) in SHRM+ eyes, respectively. Holm-adjusted within-group contrasts showed significant CRT reductions from baseline to month 6 and month 12 in both SHRM− eyes (−101.1 μm, *p* = 0.0002 and −79.1 μm, *p* = 0.0060, respectively) and SHRM+ eyes (−162.8 μm and −149.9 μm, respectively; both *p* < 0.0001). Holm-adjusted pairwise comparisons of EMMs between SHRM+ and SHRM− eyes, with the three time-point contrasts treated as a single comparison family, showed no statistically significant differences at baseline (estimated difference, +71.92 μm; 95% CI, 4.57 to 139.3; Holm-adjusted *p* = 0.110), month 6 (+10.20 μm; 95% CI, −57.15 to 77.5; Holm-adjusted *p* = 1.000), or month 12 (+1.05 μm; 95% CI, −66.30 to 68.4; Holm-adjusted *p* = 1.000). These findings indicate that CRT decreased significantly over time in both SHRM− and SHRM+ eyes, with no evidence of a statistically significant difference in CRT between SHRM groups at any individual time point after multiplicity adjustment. The non-significant time-by-SHRM interaction further indicates that the longitudinal CRT trajectory did not differ significantly according to baseline SHRM status (Figure 3C; Supplementary Table 6).

### 3.4 Multivariable and exploratory analyses of factors associated with visual outcomes

To further investigate baseline factors potentially associated with functional outcomes after Faricimab treatment, an exploratory multivariable linear regression analysis was performed using BCVA at the 12-month follow-up visit as the dependent variable. The model included baseline BCVA, age, previous anti-VEGF treatment status, and baseline SHRM presence as independent variables.

The overall regression model showed a high explanatory capacity, largely driven by the strong association between baseline BCVA and BCVA at 12 months (*R*^2^ = 0.800; adjusted *R*^2^ = 0.768; overall model *p* < 0.001). Baseline BCVA was significantly associated with BCVA at 12 months (*β* = 0.795; 95% CI 0.610–0.980; *p* < 0.001). Conversely, age was not significantly associated with final BCVA (*β* = −0.330; 95% CI −0.781–0.120; *p* = 0.143). anti-VEGF treatment status was not associated with BCVA at 12 months after adjustment (*β* = 1.115; 95% CI −6.732–8.962; *p* = 0.772). Similarly, baseline SHRM presence was not significantly associated with final BCVA (*β* = 0.380; 95% CI −6.912–7.672; *p* = 0.915).

Regression diagnostics showed no relevant violations of model assumptions. Residual diagnostics did not reveal relevant deviations from model assumptions; residual normality and homoscedasticity were supported by the Shapiro–Wilk (*p* = 0.574) and Breusch–Pagan (*p* = 0.245) tests. The maximum Cook's distance was 0.316, exceeding the conventional screening threshold of 4/n, suggesting a potentially influential observation. Therefore, a sensitivity analysis was performed by repeating the multivariable linear regression model after exclusion of the observation with the highest Cook's distance. The sensitivity analysis confirmed the robustness of the findings, with baseline BCVA remaining significantly associated with BCVA at 12 months (*β* = 0.859; 95% CI, 0.684–1.035; *p* < 0.001), whereas age (*β* = −0.128; *p* = 0.555), previous anti-VEGF treatment status (*β* = 1.048; *p* = 0.764), and baseline SHRM presence (*β* = 3.084; *p* = 0.370) remained not significantly associated with final BCVA. Detailed results of the original multivariable linear regression model and sensitivity analysis are reported in Supplementary Tables 7A,7B. In the original model, VIF values ranged from 1.15 to 1.27, indicating no relevant multicollinearity among predictors (Supplementary Table 7A). In the sensitivity analysis, the VIF for baseline BCVA increased to 4.75, whereas VIFs for the other predictors remained between 1.15 and 1.27 (Supplementary Table 7B). Although this value does not generally indicate severe multicollinearity, its increase suggests greater collinearity after exclusion of the influential observation. The model showed good explanatory capacity, but these estimates should be interpreted cautiously given the exploratory nature of the analysis, the limited sample size, and the number of covariates included.

Overall, these exploratory findings suggest that baseline BCVA showed the strongest association with 12-month BCVA in this exploratory multivariable model in this real-world cohort, whereas anti-VEGF treatment status, age, and baseline SHRM presence were not independently associated with final BCVA after adjustment. Given the limited sample size, these findings should be interpreted as hypothesis-generating rather than confirmatory.

## 4 Discussion

In this study, we analyzed the real-world anatomical and functional outcomes of a cohort of 30 patients (30 eyes) with nAMD observed during a one-year follow-up of Faricimab therapy. We included treatment-naïve eyes and eyes previously treated with intravitreal Aflibercept. The entire cohort showed a significant BCVA improvement compared to baseline at 12-month follow-up. Although the retrospective before-after design and the absence of a comparator group preclude causal conclusions, these findings demonstrate a significant longitudinal improvement in BCVA following Faricimab initiation; however, the uncontrolled retrospective design precludes attribution of this improvement specifically to treatment. Longitudinal analysis using LMMs confirmed a significant effect of time on BCVA, with statistically significant improvements already observed at month 6 and with BCVA remaining significantly higher than baseline at month 12. Although numerical differences in visual gain were observed between treatment-naïve and non-naïve eyes, no significant time-by-treatment-history interaction was detected, indicating no statistically significant evidence that previous anti-VEGF treatment modified BCVA trajectories in this cohort. These functional outcomes were accompanied by a significant longitudinal reduction in CRT. Although exploratory within-group analyses showed significant CRT reductions in naïve eyes at 6 and 12 months, the LMM evaluating longitudinal trajectories did not demonstrate a significant time-by-treatment-history interaction. Therefore, these within-group findings should not be interpreted as evidence of differential anatomical response between naïve and non-naïve eyes.

Differently from a Pro Re Nata (PRN) approach, which is based on treating the patient once disease activity is detected, TAE sets out to manage disease activity through a proactive approach based on two different phases: the loading phase, which is the first one and is based on a fixed number of monthly injections, followed by a maintenance phase, which is based on further injections whose timing is progressively lengthened up to a maximum interval, in order to maintain disease stability, rather than treating the patient once disease reactivation has already happened.

TAE was first introduced by Spaide et al. in 2007 (36) and was subsequently validated by multiple trials in neovascular-AMD [TREX-AMD trial, 2018 (37); CANTREAT trial, 2019 (38); AZURE trial, 2024 (39)] and further settings such as DME (RETAIN study). Previous randomized and real-world studies have reported that TAE may help maintain stable visual outcomes while preventing disease reactivation through proactive treatment strategies and may reduce patient burden (in terms of office visits and injections) and healthcare burden compared with PRN regimens (37, 40). TAE has also been associated with patient-tailored treatment intervals and favorable visual outcomes while potentially reducing treatment burden compared with fixed regimens (38, 39).

Patients received four monthly loading injections, followed by a treat-and-extend regimen, according to the treatment protocol adopted at our centers (41), and were subsequently retreated in the maintenance phase with increasing treatment intervals up until the longest possible interval achieved by each patient, with a maximum retreatment interval of 16 weeks between two consecutive injections (Q16w) and shorter intervals being 12 weeks (Q12w) and 8 weeks (Q8w).

Overall, 19/30 eyes (63.3%) reached and maintained a Q16w interval during follow-up, including 14/22 treatment-naïve eyes (63.6%). These extended dosing intervals indicate that a proportion of patients maintained longer treatment intervals during follow-up; however, the impact on treatment burden cannot be established without a comparator group or formal evaluation of healthcare utilization. As reported by Panos et al. in their review on clinical studies regarding Faricimab as a novel long-acting anti-VEGF agent (42), 78% of naïve eyes enrolled in the pivotal Phase III clinical trials on Faricimab (TENAYA and LUCERNE) managed to reach and maintain a Q12w regimen, whereas 45% reached and maintained the longest possible interval (Q16w) by year 1, achieving visual, morphological and functional outcomes non-inferior to Aflibercept. These extended dosing intervals suggest the potential for reduced injection frequency; however, because our study did not include a comparator group or a formal within-patient comparison with previous treatment regimens, no definitive conclusions regarding treatment-burden reduction can be drawn.

The prespecified primary functional endpoint of the study was BCVA. The LMM demonstrated a significant overall effect of time on visual outcomes, with statistically significant BCVA improvements observed both at month 6 and month 12 compared with baseline. The use of LMM allowed evaluation of the entire longitudinal trajectory rather than isolated comparisons at individual time points. The absence of a significant time-by-treatment-history interaction suggests that there was no statistical evidence that previous anti-VEGF exposure influenced longitudinal BCVA trajectories following Faricimab treatment in this cohort. Notably, our cohort of patients encompassed different types of retinal fluid according to their distribution (that is, at the intraretinal, subretinal and/or sub-RPE level); however, in our exploratory analysis, baseline retinal fluid distribution was not associated with statistically detectable differences in BCVA changes during follow-up. A similar observation was previously reported in our real-world study evaluating another anti-VEGF agent, Brolucizumab (35). However, these findings should be interpreted with caution because fluid categories were not mutually exclusive and subgroup sizes were limited, reducing the statistical power to detect potential differences among specific fluid patterns. Therefore, larger studies with predefined analyses and adequate sample sizes are required to determine whether baseline retinal fluid characteristics may influence functional outcomes after Faricimab treatment. In parallel, longitudinal LMM analysis demonstrated a significant decrease in CRT at both month 6 and month 12, supporting the observed anatomical response over the 12-month follow-up period. Although CRT reduction was numerically greater among naïve eyes, the longitudinal model did not demonstrate a statistically significant difference in anatomical response according to anti-VEGF treatment status. Overall, these findings indicate a significant longitudinal anatomical improvement after Faricimab initiation in this real-world cohort. These observations are consistent with the currently available literature (42, 43) and suggest that Faricimab may represent a potential therapeutic option warranting further investigation in patients with persistent or suboptimal response to previous anti-VEGF therapy.

Another interesting feature which was analyzed in our study concerns SHRM, an OCT biomarker found in many pathological conditions involving the retina, such as age-related macular degeneration, pachychoroid spectrum disorders, pathological myopia and more. As described by Feo et al. in their review (44), SHRM is a finding characterized by the presence of hyperreflective material at the subretinal level, whose composition is rather heterogeneous and includes fibrinous, exudative and drusenoid material, as well as hemorrhage and neovascularization. In the setting of AMD, SHRM has been broadly confirmed to be characterized by a negative prognostic value, being linked to worse VA both at baseline and during follow-up, as well as a higher risk of subretinal fibrosis and scarring: this is hypothesized to be explained by the mechanical separation of the RPE and choriocapillaris from the overlying neuroretina, which in turn causes a significant decrease in both oxygen and nutrient supply to the outer retina, with subsequent photoreceptor loss. However, it is important to consider that the prognostic impact of SHRM may be influenced by several morphological and anatomical features, including margin morphology, reflectivity, overall size and thickness, as well as the specific MNV subtype associated with SHRM. These characteristics were not separately analyzed in our cohort and may represent relevant factors for future investigations.

Our study allows for some interesting considerations regarding SHRM. The LMM showed a statistically significant time-by-baseline SHRM status interaction for BCVA, suggesting that the longitudinal pattern of visual change over follow-up differed according to baseline SHRM status. However, multiplicity-adjusted pairwise comparisons did not demonstrate statistically significant between-group differences at individual time points. Thus, the significant interaction should be interpreted as evidence of different overall longitudinal trajectories rather than as evidence of a significant between-group difference at any specific follow-up visit. Although the estimated longitudinal changes appeared numerically different between SHRM groups, multiplicity-adjusted comparisons did not demonstrate significant between-group differences at any individual time point. SHRM-positive eyes also had numerically lower baseline BCVA than SHRM-negative eyes, although this difference was not statistically significant and was no longer apparent at month 12. In contrast, the anatomical LMM analysis did not demonstrate a statistically significant time-by-SHRM interaction for CRT, suggesting that baseline SHRM status was not associated with a statistically detectable difference in the longitudinal evolution of CRT. SHRM thickness also decreased significantly at months 6 and 12; however, this analysis was exploratory and was intended to describe SHRM evolution over time rather than to assess SHRM thickness reduction as a predictor of treatment response. Given the exploratory nature and limited sample size, these findings should be interpreted cautiously and do not establish a clinically meaningful differential response according to SHRM status. The biological mechanisms underlying SHRM evolution remain unclear. Given the heterogeneous composition of SHRM, including fibrovascular, fibrinous, exudative, and hemorrhagic components, different structural components may undergo variable changes during follow-up. Indeed, Feo et al. highlighted the importance of evaluating specific SHRM features by means of multimodal imaging and contrast-enhanced OCT to better characterize their potential relationship with treatment response (44).

Our SHRM results should be interpreted with caution, considering the relatively limited sample size of our study, which may have reduced the statistical power to detect potential differences between SHRM + and SHRM- groups. Importantly, the significant time-by-SHRM interaction observed for BCVA should not be equated with a significant between-group difference at individual time points, as no statistically significant differences were identified in the multiplicity-adjusted pairwise comparisons. Conversely, the absence of a significant time-by-SHRM interaction for CRT should be interpreted as the absence of a statistically detectable difference in longitudinal anatomical trajectories, rather than as evidence of equivalence between SHRM-positive and SHRM-negative eyes. Furthermore, because the study was exploratory and based on a relatively small cohort, the observed time-by-SHRM interaction for BCVA should be considered hypothesis-generating and requires confirmation in larger cohorts. These findings can be considered in the context of the recent study by Yanai et al. (45), who evaluated nAMD patients treated with Faricimab over a 6-month follow-up period. In that study, SHRM-positive eyes had lower VA at baseline and at month 6, although no significant association was found between SHRM or other OCT biomarkers and final VA. Interestingly, SHRM, as well as IRF and SMH, was associated with greater CRT improvement, but none of these OCT biomarkers was significantly associated with final CRT. These findings are broadly consistent with our observation that baseline SHRM status was not associated with statistically significant differences in BCVA or CRT at individual follow-up time points after multiplicity adjustment, despite the significant time-by-SHRM interaction observed for BCVA. However, direct comparisons between the two studies should be made cautiously because of differences in study design, follow-up duration, sample characteristics, and statistical approaches. In another interesting study, Sacconi et al. (46) reported no correlation between SHRM and final VA in patients diagnosed with Type 3 MNV and treated with anti-VEGF injections. These findings highlight the importance of considering the specific MNV subtype associated with SHRM, as it may influence visual outcomes. Lastly, none of the previous studies analyzed in detail the SHRM thickness and its change over time. Moreover, it is important to emphasize that our study did not include mechanistic analyses, SHRM composition was not directly characterized, and therefore no definitive conclusions regarding the biological nature of SHRM or its differential response to treatment can be drawn. On the other hand, most available evidence regarding SHRM in nAMD comes from studies involving anti-VEGF agents other than Faricimab (including bevacizumab, ranibizumab, and aflibercept). Whether Ang-2/VEGF dual inhibition may influence SHRM evolution remains unknown and cannot be determined from our data, as SHRM composition and related angiogenic or inflammatory pathways were not directly investigated.

Exploratory multivariable analysis also provided additional insights into potential predictors of visual outcome after Faricimab treatment. In our cohort, baseline BCVA remained significantly associated with BCVA at 12 months, whereas age, previous anti-VEGF exposure, and baseline SHRM presence were not significantly associated with final visual acuity after adjustment. These findings suggest that baseline functional status may represent an important determinant of final visual outcome; however, they should be interpreted cautiously. Given the limited sample size and exploratory nature of this analysis, these findings should be interpreted as hypothesis-generating and require validation in larger cohorts.

Finally, a key point to consider is the possibility of differences in treatment response among different MNV subtypes. Differences in treatment response have been described among the different MNV subtypes, although their magnitude and clinical relevance remain debated (47). Type 1 MNV, due to its sub-RPE location, has been hypothesized to show a less favorable response to anti-VEGF therapy because limited drug penetration toward the neovascular complex may reduce treatment efficacy and potentially require a higher number of injections to achieve disease control. Conversely, type 2 MNV because of its subretinal extension, may be more directly exposed to anti-VEGF agents, which could contribute to a more favorable anatomical response. Type 3 MNV has also been associated with a favorable anatomical response to anti-VEGF therapy; however, visual outcomes may vary depending on disease stage, the presence of associated atrophic changes, and the extent of neuroretinal damage. However, MNV1 was also reported to be the MNV type most likely associated with VA preservation over time, while MNV2 was associated with a significantly higher risk of subretinal fibrosis and scarring and MNV3 was associated with a significantly higher risk of geographical atrophy. In our cohort, exploratory analyses did not detect statistically significant differences in final BCVA or CRT according to baseline MNV subtype. However, the present study was not designed or powered to evaluate whether MNV subtype is associated with differential anatomical or functional outcomes after Faricimab treatment; therefore, these findings should be interpreted cautiously, particularly considering the limited number of eyes within each subgroup and the very small type 3 MNV subgroup (*N* = 2). The absence of statistically significant differences should not be interpreted as evidence that MNV subtype is not associated with differences in clinical outcomes, but rather as the absence of a statistically detectable association within this small real-world cohort. These findings should be considered hypothesis-generating and require confirmation in larger studies with longer follow-up periods.

No systemic adverse events and only one ocular adverse event were observed during follow-up. However, given the limited sample size and the observational design of the study, these findings should be interpreted cautiously and do not allow definitive conclusions regarding the overall safety profile of Faricimab, particularly with regard to uncommon adverse events. One ocular adverse event occurred during follow-up, consisting of vitreitis associated with elevated intraocular pressure following intravitreal treatment, which was subsequently managed with pars plana vitrectomy. No further adverse events were observed during the study period; however, the limited cohort size precludes meaningful assessment of rare treatment-related complications.

Although, to our knowledge, this is the first two-center Italian study reporting 12-month real-world outcomes, the main limitation is the relatively small sample size, particularly within subgroup analyses. Therefore, the statistical power to detect differences between subgroups was limited, and non-significant findings should not be interpreted as evidence of equivalence. In addition, the adoption of LMMs allowed appropriate handling of repeated measurements from the same eye; although mixed-effects modelling provides an appropriate framework for longitudinal ophthalmological data, the limited sample size may have reduced the ability to detect small interaction effects, particularly in subgroup analyses. Therefore, non-significant interaction terms should be interpreted as an absence of statistically detectable differences rather than proof of equivalence between subgroups. Moreover, given the exploratory real-world nature of the study, the analyses were primarily aimed at generating clinically relevant hypotheses rather than providing confirmatory evidence. Although BCVA was prespecified as the primary functional endpoint, CRT was evaluated as a key secondary anatomical outcome reflecting morphological response. Given the exploratory real-world design of the study, additional secondary, subgroup, and biomarker analyses were considered hypothesis-generating. Although Tukey correction was applied to pairwise comparisons derived from EMMs, no global multiplicity correction was applied across all exploratory analyses. Therefore, the possibility of an increased risk of type I error due to multiple comparisons cannot be excluded, and findings derived from these analyses should be interpreted cautiously and require confirmation in larger prospective studies with prespecified statistical frameworks and appropriate control of multiplicity. Moreover, the retrospective design and the inclusion of patients from two centers may have introduced potential referral and selection biases. In particular, our study population consisted only of patients with active nAMD who were considered eligible for Faricimab treatment according to predefined inclusion and exclusion criteria. Although these criteria were necessary to obtain a homogeneous cohort and minimize potential confounding factors, they may have resulted in the selection of a specific subgroup of patients that may not fully reflect the broader nAMD population encountered in routine clinical practice. Therefore, the external generalizability of our findings may be limited. Furthermore, differences between included and excluded patients could not be assessed, as clinical data from patients who did not meet the eligibility criteria were not systematically collected. Finally, our study did not include a comparator group and injection frequency was not compared with previous therapies in switched patients. Therefore, any potential reduction in treatment burden can only be inferred from the dosing intervals achieved, since no formal assessment of patient or healthcare burden was performed. In addition, the relatively short follow-up period and limited sample size prevent definitive conclusions regarding the long-term durability of Faricimab treatment, the evolution of structural biomarkers, and the potential differential response according to MNV characteristics. Future prospective multicenter studies with larger cohorts, longer follow-up periods, and predefined statistical analysis plans are warranted to confirm these preliminary real-world observations.

In conclusion, in this real-world observational cohort, BCVA and CRT improved significantly over 12 months following initiation of Faricimab treatment. No statistically significant differences in longitudinal trajectories according to previous anti-VEGF exposure were detected. However, given the retrospective uncontrolled design, limited sample size, and absence of a comparator group, these findings should be considered hypothesis-generating and interpreted as supportive of further prospective comparative studies rather than as definitive evidence of treatment efficacy or safety. Exploratory analyses investigated potential associations between SHRM evolution, baseline anatomical characteristics, and observed outcomes; however, these findings remain hypothesis-generating and require validation in larger cohorts.

## Data Availability

The original contributions presented in the study are included in the article/Supplementary Material, further inquiries can be directed to the corresponding author/s.
