# Supplementary material for: Real-world anatomical and functional outcomes following Faricimab therapy in neovascular age-related macular degeneration: a one-year observational study
Source: Front Med (Lausanne). 2026 Sep 28;13:1901385. doi: 10.3389/fmed.2026.1901385 (PMC13654786; doi:10.3389/fmed.2026.1901385)
Supplement: Supplementary file 1 [file Supplementaryfile1.docx]

Supplementary Material

# 1 Supplementary Tables

**Supplementary Table 1**

| **Fixed effect** | **Estimate** | **SE** | **df** | **95% CI** | **Model-based p-value** |
| --- | --- | --- | --- | --- | --- |
| Intercept (Baseline) | 40.50 | 3.29 | 32.9 | 33.81 to 47.19 | < 0.001 |
| Month 6 vs Baseline | +7.10 | 1.42 | 58.0 | 4.26 to 9.94 | < 0.001 |
| Month 12 vs Baseline | +6.67 | 1.42 | 58.0 | 3.83 to 9.51 | < 0.001 |
| **Random effect** | **Variance** |  |  |  |  |
| Eye-level random intercept | 294.31 |  |  |  |  |
| Residual | 30.26 |  |  |  |  |

**Supplementary Table 1.** Model coefficients from the linear mixed-effects model evaluating longitudinal changes in BCVA (N = 30). Estimates are presented with standard errors (SE), degrees of freedom (df), 95% confidence intervals (CI), and model-based p-values for the corresponding fixed effects. 95% CIs and p-values are unadjusted for multiple comparisons. Pairwise contrasts between estimated marginal means were performed separately using Tukey’s method for multiplicity adjustment; these adjusted contrasts are not shown in this table and are reported in the main text. Random-effect variances for the eye-level intercept and residual error are reported below.

**Supplementary Table 2A**

| **Fixed effect** | **Estimate** | **SE** | **df** | **95% CI** | **Model-based p-value** |
| --- | --- | --- | --- | --- | --- |
| Intercept (Baseline, Non-naïve) | 36.38 | 6.39 | 31.83 | 23.35 to 49.40 | <0.001 |
| Month 6 vs Baseline | 5.38 | 2.78 | 56.00 | -0.20 to 10.95 | 0.0586 |
| Month 12 vs Baseline | 6.38 | 2.78 | 56.00 | 0.80 to 11.95 | 0.0258 |
| Naïve vs Non-naïve | 5.63 | 7.47 | 31.83 | -9.58 to 20.83 | 0.4567 |
| Month 6 × Naïve status | 2.35 | 3.25 | 56.00 | -4.16 to 8.87 | 0.4724 |
| Month 12 × Naïve status | 0.40 | 3.25 | 56.00 | -6.12 to 6.91 | 0.9031 |
| **Omnibus Type III tests** | **df** | **F** | **Omnibus p-value** |  |  |
| Time | 2, 56 | 3.033 | 0.056 |  |  |
| Treatment history | 1, 28 | 0.820 | 0.373 |  |  |
| Time × Treatment History | 2, 56 | 0.300 | 0.742 |  |  |

**Supplementary Table 2A.** Model coefficients from the linear mixed-effects model assessing longitudinal BCVA changes according to previous anti-VEGF treatment status (N = 30). Fixed-effect estimates are reported as regression coefficients with standard errors (SE), degrees of freedom (df), 95% confidence intervals (CIs), and model-based p-values. Omnibus p-values were obtained from Type III analysis of variance with Kenward–Roger degrees-of-freedom adjustment. The reference categories for the fixed effects were baseline for time and non-naïve status for treatment history. Pairwise estimated marginal mean contrasts between treatment-naïve and non-naïve eyes at baseline, month 6, and month 12 were treated as a single comparison family and jointly adjusted for multiple comparisons using Holm’s method; Holm-adjusted p-values are reported in the main text, whereas the corresponding 95% CIs are unadjusted. The 95% CIs and p-values reported for the fixed effects in this table are unadjusted for multiple comparisons. The model included time, previous anti-VEGF treatment status, and the time-by-treatment-history interaction as fixed effects, with eye identification included as a random intercept.

**Supplementary Table 2B**

| **Treatment history** | **Baseline** | **Month 6** | **Month 12** |
| --- | --- | --- | --- |
| Naïve | 42.0  (95% CI: 34.1–49.9) | 49.7  (95% CI: 41.9–57.6) | 48.8  (95% CI: 40.9–56.6) |
| Non-naïve | 36.4  (95% CI: 23.4–49.4) | 41.8  (95% CI: 28.7–54.8) | 42.8  (95% CI: 29.7–55.8) |
| **Random effect** | **Variance** |  |  |
| Eye-level random intercept | 295.96 |  |  |
| Residual | 31.01 |  |  |

**Supplementary Table 2B.** Estimated marginal means from the linear mixed-effects model assessing longitudinal BCVA changes according to previous anti-VEGF treatment status (N = 30). Estimated marginal means are reported as BCVA values with model-based 95% confidence intervals (CIs) at each follow-up time point. The reported 95% CIs are unadjusted for multiple comparisons. The model included time, previous anti-VEGF treatment status, and the time-by-treatment-history interaction as fixed effects, with eye identification included as a random intercept. Random-effect estimates are reported as variance components for the eye-level random intercept and residual error. Pairwise contrasts between estimated marginal means of treatment-naïve and non-naïve eyes were treated as a single comparison family across baseline, month 6, and month 12 and jointly adjusted for multiple comparisons using Holm’s method; Holm-adjusted p-values are reported in the main text, whereas the corresponding 95% CIs are unadjusted.

**Supplementary Table 3**

| **Fixed effect** | **Estimate** | **SE** | **df** | **95% CI** | **Model-based p-value** |
| --- | --- | --- | --- | --- | --- |
| Intercept (Baseline) | 372.0 | 17.0 | 67.9 | 338.08 to 405.92 | < 0.001 |
| Month 6 vs Baseline | -130.0 | 19.0 | 58.0 | -168.03 to -91.97 | < 0.001 |
| Month 12 vs Baseline | -112.0 | 19.0 | 58.0 | -150.03 to -73.97 | < 0.001 |
| **Random effect** | **Variance** |  |  |  |  |
| Eye-level random intercept | 3246.23 |  |  |  |  |
| Residual | 5403.19 |  |  |  |  |

**Supplementary Table 3**. Model coefficients from the linear mixed-effects model evaluating longitudinal changes in CRT (N = 30). Estimates are presented with standard errors (SE), degrees of freedom (df), 95% confidence intervals (CIs), and model-based p-values for the corresponding fixed effects. The reported CIs and p-values refer to the model-based fixed-effect coefficients and are unadjusted for multiple comparisons. Pairwise contrasts between estimated marginal means (EMMs) were performed separately using Sidak adjustment for multiple comparisons and are reported in the main text where applicable. Random-effect variances for the eye-level intercept and residual error are reported below.

**Supplementary Table 4A**

| **Fixed effect** | **Estimate** | **SE** | **df** | **95% CI** | **Model-based p-value** |
| --- | --- | --- | --- | --- | --- |
| Intercept (Baseline, Non-naïve) | 327.25 | 32.92 | 63.99 | 261.48 to 393.02 | <0.001 |
| Month 6 vs Baseline | -71.13 | 36.20 | 56.00 | -143.65 to 1.39 | 0.0544 |
| Month 12 vs Baseline | -71.25 | 36.20 | 56.00 | -143.77 to 1.27 | 0.0540 |
| Naïve vs Non-naïve | 61.02 | 38.44 | 63.99 | -15.70 to 137.80 | 0.1174 |
| Month 6 × Naïve status | -80.10 | 42.28 | 56.00 | -164.79 to 4.59 | 0.0633 |
| Month 12 × Naïve status | -55.75 | 42.28 | 56.00 | -140.44 to 28.94 | 0.1926 |
| **Omnibus Type III tests** | **df** | **F** | **Omnibus p-value** |  |  |
| Time | 2, 56 | 16.659 | < 0.0001 |  |  |
| Treatment history | 1, 28 | 0.281 | 0.600 |  |  |
| Time × Treatment history | 2, 56 | 1.887 | 0.161 |  |  |

**Supplementary Table 4A.** Model coefficients from the linear mixed-effects model assessing longitudinal CRT changes according to previous anti-VEGF treatment status (N = 30). Fixed-effect estimates are reported as regression coefficients with standard errors (SE), degrees of freedom (df), 95% confidence intervals (CIs), and model-based p-values. Omnibus p-values were obtained from Type III analysis of variance with Kenward–Roger degrees-of-freedom adjustment. The reference categories for the fixed effects were baseline for time and non-naïve status for treatment history. Exploratory within-group estimated marginal mean contrasts between baseline and month 6 and between baseline and month 12 in treatment-naïve eyes were treated as a single comparison family and jointly adjusted for multiple comparisons using Holm’s method; these adjusted contrasts are reported in the main text and are not shown in this table. The 95% CIs and p-values reported for the fixed effects are unadjusted for multiple comparisons. The model included time, previous anti-VEGF treatment status, and the time-by-treatment-history interaction as fixed effects, with eye identification included as a random intercept.

**Supplementary Table 4B**

| **Treatment history** | **Baseline** | **Month 6** | **Month 12** |
| --- | --- | --- | --- |
| Naïve | 388  (95% CI: 349–428) | 237  (95% CI: 197–277) | 261  (95% CI: 222–301) |
| Non-naïve | 327  (95% CI: 261–393) | 256  (95% CI: 190–322) | 256  (95% CI: 190–322) |
| **Random effect** | **Variance** |  |  |
| Eye-level random intercept | 3428 |  |  |
| Residual | 5243 |  |  |

**Supplementary Table 4B.** Estimated marginal means from the linear mixed-effects model assessing longitudinal CRT changes according to previous anti-VEGF treatment status (N = 30). Estimated marginal means are reported as CRT values with model-based 95% confidence intervals (CIs) at each follow-up time point. The reported 95% CIs are unadjusted for multiple comparisons. The model included time, previous anti-VEGF treatment status, and the time-by-treatment-history interaction as fixed effects, with eye identification included as a random intercept. Random-effect estimates are reported as variance components for the eye-level random intercept and residual error. Exploratory within-group estimated marginal mean contrasts between baseline and month 6 and between baseline and month 12 in treatment-naïve eyes were treated as a single comparison family and jointly adjusted for multiple comparisons using Holm’s method; these adjusted contrasts are not shown in this table and the Holm-adjusted p-values are reported in the main text, whereas the corresponding 95% CIs are unadjusted.

**Supplementary Table 5**

| **Fixed effect** | **Estimate** | **SE** | **df** | **95% CI** | **Coefficient- level p-value** |
| --- | --- | --- | --- | --- | --- |
| Intercept (SHRM− Baseline) | 44.375 | 4.536 | 31.4 | 35.13 to 53.62 | < 0.001 |
| Month 6 vs Baseline (SHRM−) | +4.313 | 1.869 | 56.0 | 0.57 to 8.06 | 0.0247 |
| Month 12 vs Baseline (SHRM−) | +3.688 | 1.869 | 56.0 | -0.06 to 7.43 | 0.0534 |
| SHRM+ vs SHRM− at baseline | -8.304 | 6.640 | 31.4 | -21.84 to 5.23 | 0.2203 |
| Month 6 × SHRM interaction | +5.973 | 2.735 | 56.0 | 0.49 to 11.45 | 0.0332 |
| Month 12 × SHRM interaction | +6.384 | 2.735 | 56.0 | 0.90 to 11.86 | 0.0232 |
| **Omnibus Type III tests** | **df** | **F** | **Omnibus p-value** |  |  |
| Time | 2, 56 | 17.96 | < 0.001 |  |  |
| SHRM status | 1, 28 | 0.421 | 0.522 |  |  |
| Time × SHRM status | 2, 56 | 3.413 | 0.040 |  |  |
| **Random effect** | **Variance** |  |  |  |  |
| Eye-level random intercept | 301.28 |  |  |  |  |
| Residual | 27.93 |  |  |  |  |

**Supplementary Table 5**. Model coefficients, omnibus Type III tests, and random-effect variances from the linear mixed-effects model evaluating the association between baseline SHRM status (SHRM-positive eyes: SHRM+, N = 14; SHRM-negative eyes: SHRM−, N = 16) and longitudinal changes in best-corrected visual acuity (BCVA). The model included time, baseline SHRM status, and the time-by-SHRM interaction as fixed effects, with eye identification included as a random intercept. Fixed-effect estimates are reported with standard errors (SE), Kenward–Roger degrees of freedom (df), 95% confidence intervals (CI), and coefficient-level p-values. Omnibus p-values were obtained from Type III analysis of variance with Kenward–Roger degrees-of-freedom adjustment. The reference categories were SHRM− and baseline. Post hoc estimated marginal mean contrasts were adjusted for multiple comparisons using Holm’s method. Within-group contrasts between baseline and month 6 and between baseline and month 12 were adjusted within each SHRM-status group, whereas the three between-group contrasts (SHRM+ vs SHRM−) at baseline, month 6, and month 12 were treated as a single comparison family and jointly adjusted using Holm’s method. These adjusted EMM contrasts are reported in the main text and are not shown in this table. Random-effect variances for the eye-level random intercept and residual error are reported below the fixed-effects and omnibus test results. SHRM, subretinal hyperreflective material; BCVA, best-corrected visual acuity; SE, standard error.

**Supplementary Table 6**

| **Fixed effect** | **Estimate** | **SE** | **df** | **Coefficient- level p-value** | **95% CI** |
| --- | --- | --- | --- | --- | --- |
| Intercept (SHRM− Baseline) | 338.44 | 23.03 | 64.6 | < 0.001 | 292.44 to 384.44 |
| Month 6 vs Baseline (SHRM−) | -101.06 | 25.49 | 56.0 | < 0.001 | -152.13 to -49.99 |
| Month 12 vs Baseline (SHRM−) | -79.06 | 25.49 | 56.0 | 0.003 | -130.13 to -27.99 |
| SHRM+ vs SHRM− at Baseline | +71.92 | 33.72 | 64.6 | 0.0367 | 4.57 to 139.27 |
| Month 6 × SHRM interaction | -61.72 | 37.32 | 56.0 | 0.1038 | -136.49 to 13.04 |
| Month 12 × SHRM interaction | -70.87 | 37.32 | 56.0 | 0.0627 | -145.63 to 3.90 |
| **Omnibus Type III tests** | **df** | **F** | **Omnibus p-value** |  |  |
| Time | 2, 56 | 29.50 | < 0.001 |  |  |
| SHRM status | 1, 28 | 1.143 | 0.294 |  |  |
| Time × SHRM status | 2, 56 | 2.134 | 0.128 |  |  |
| **Random effect** | **Variance** |  |  |  |  |
| Eye-level random intercept | 3289 |  |  |  |  |
| Residual | 5200 |  |  |  |  |

**Supplementary Table 6.** Model coefficients, omnibus Type III tests, and random-effect variances from the linear mixed-effects model evaluating the association between baseline SHRM status (SHRM-positive eyes: SHRM+, N = 14; SHRM-negative eyes: SHRM−, N = 16) and longitudinal changes in central retinal thickness (CRT). The model included time, baseline SHRM status, and the time-by-SHRM interaction as fixed effects, with eye identification included as a random intercept. Fixed-effect estimates are reported with standard errors (SE), Kenward–Roger degrees of freedom (df), 95% confidence intervals (CI), and coefficient-level p-values. Omnibus p-values were obtained from Type III analysis of variance with Kenward–Roger degrees-of-freedom adjustment. The reference categories were SHRM− and baseline. Post hoc estimated marginal mean contrasts were adjusted for multiple comparisons using Holm’s method. Within-group contrasts between baseline and month 6 and between baseline and month 12 were adjusted within each SHRM-status group, whereas the three between-group contrasts (SHRM+ vs SHRM−) at baseline, month 6, and month 12 were treated as a single comparison family and jointly adjusted using Holm’s method. These adjusted EMM contrasts are reported in the main text and are not shown in this table. Random-effect variances for the eye-level random intercept and residual error are reported below the fixed-effects and omnibus test results. SHRM, subretinal hyperreflective material; CRT, central retinal thickness; SE, standard error.

**Supplementary Table 7A**

| **Predictor** | **β** | **95% CI** | **p-value** | **VIF** |
| --- | --- | --- | --- | --- |
| Baseline BCVA | 0.795 | 0.610–0.980 | < 0.001 | 1.15 |
| Age | -0.330 | -0.781–0.120 | 0.143 | 1.15 |
| Previous anti-VEGF | 1.115 | -6.732–8.962 | 0.772 | 1.15 |
| SHRM presence | 0.380 | -6.912–7.672 | 0.915 | 1.27 |
| Model summary: R²=0.800; adjusted R²=0.768; overall model p < 0.001 | | | | |

**Supplementary Table 7A**. Multivariable linear regression coefficients for factors associated with best-corrected visual acuity at 12 months after Faricimab treatment (N=30). Regression coefficients (β), 95% confidence intervals (CI), p-values, and variance inflation factors (VIF) are reported for each predictor included in the multivariable model. VIF values are reported to assess potential collinearity among predictors. Model performance was summarized by the coefficient of determination (R²=0.800), adjusted R²=0.768, and overall model significance (p < 0.001). BCVA, best-corrected visual acuity; CI, confidence interval; anti-VEGF, anti-vascular endothelial growth factor; SHRM, subretinal hyperreflective material; VIF, variance inflation factor; β, regression coefficient; R², coefficient of determination.

**Supplementary Table 7B**

| **Predictor** | **β** | **95% CI** | **p-value** | **VIF** |
| --- | --- | --- | --- | --- |
| Baseline BCVA | 0.859 | 0.684–1.035 | < 0.001 | 4.75 |
| Age | -0.128 | -0.568–0.313 | 0.555 | 1.26 |
| Previous anti-VEGF | 1.048 | -6.068–8.165 | 0.764 | 1.15 |
| SHRM presence | 3.084 | -3.880–10.048 | 0.370 | 1.27 |
| Sensitivity analysis excluding influential observation (Cook’s distance = 0.316): R²=0.842; adjusted R²=0.815; overall model p < 0.001. | | | | |

**Supplementary Table 7B**. Sensitivity analysis of the multivariable linear regression coefficients for factors associated with best-corrected visual acuity at 12 months after Faricimab treatment (N=29). The sensitivity analysis was performed after exclusion of the influential observation identified by Cook’s distance (0.316). Regression coefficients (β), 95% confidence intervals (CI), p-values, and variance inflation factors (VIF) are reported. The sensitivity model showed improved explanatory performance (R²=0.842; adjusted R²=0.815; overall model p < 0.001). The VIF for baseline BCVA increased to 4.75 after exclusion of the influential observation, whereas VIFs for the other predictors remained between 1.15 and 1.27. BCVA, best-corrected visual acuity; CI, confidence interval; anti-VEGF, anti-vascular endothelial growth factor; SHRM, subretinal hyperreflective material; VIF, variance inflation factor; β, regression coefficient; R², coefficient of determination.
